# Prevalence of Parent-Reported Food Allergies and Associated Risk Predictors Among Children in Saudi Arabia

**DOI:** 10.7759/cureus.33974

**Published:** 2023-01-19

**Authors:** Ahmad Alzahrani, Sara Alrebaiee, Shmookh Alsalmi, Mazen Althomali, Rabeah Alsofyani, Faisal Alkhudaydi, Mugtaba Osman

**Affiliations:** 1 Department of Pediatrics, College of Medicine, Taif University, Taif, SAU; 2 Department of Internal Medicine, King Faisal Medical Complex, Taif, SAU; 3 Department of Pediatrics, Taif University, Taif, SAU; 4 Department of Pediatrics, Children's Hospital, Taif, SAU; 5 Department of Family Medicine, Joint Program of Family Medicine, Jeddah, SAU; 6 Department of Psychiatry, Armed Forces Center for Psychiatric Care, Taif, SAU

**Keywords:** atopic dermatitis, asthma, children, saudi arabia, prevalence, food allergy

## Abstract

Background and objective: The worldwide prevalence of food allergies has been increasing recently. Due to limited data on food allergy (FA) in Saudi Arabia, this study was conducted to estimate the prevalence and risk factors of parent-reported food allergies and clinical manifestations in children in Taif, Saudi Arabia.

Methodology: A cross-sectional questionnaire-based observational study was conducted from July 2019 to December 2020. A total of 508 parents of school children (aged five to eight years) responded to the questions based on the child’s health and food allergies.

Results: FA (16.1%) was observed as the most common type of allergy among children. The prevalence of other atopic diseases was estimated at 30.5%. The most common allergy-causing foods were eggs (4.9%), peanuts (2.7%), and sesame (2.5%). Rash, itching without rash, and vomiting were the most common FA symptoms. The presence of childhood eczema (p< 0.0001), allergic rhinitis (p= 0.005), and the father’s history of allergy (p= 0.005) were all significant and independent predictors/risk factors for FA among the studied children.

Conclusion: We noted substantial parents’ concern with food allergies among children in Saudi Arabia, which necessitates the establishment of effective diagnosis and treatment strategies and primary prevention initiatives.

## Introduction

Food allergy (FA), according to the National Institute of Allergy and Infectious Diseases, may be defined as “an adverse health effect arising from a specific immune response that occurs reproducibly on exposure to a given food” [1]. Childhood FA is a significant public health concern [2,3] and may have detrimental or potentially life-threatening clinical outcomes [4], substantially impacting the quality of life, social status, and mental state of the affected children and their families [5,6]. It is also associated with a high financial burden with an estimated economic cost of $4184 per year per child [7].

Manifestations of immunoglobulin E (IgE)-mediated FA vary and, depending on the intensity, they may range from cutaneous symptoms, such as pruritus, urticaria, and angioedema, to severe symptoms in the form of anaphylaxis involving multiple systems, including the respiratory, gastrointestinal, or even cardiovascular systems; in rare cases, it can be life-threatening, requiring hospitalization [8]. The severity of FA is associated with the degree of food exposure or the amount of food ingested, its digestion, and epithelial permeability [9]. Fortunately, in children, many FAs such as milk, egg, and wheat resolve as they grow up; however, in some cases, they may persist lifelong, as in the case of peanuts, tree nuts, and fish [10]. Most FAs in childhood include allergies to milk, eggs, wheat, fish, soy, and peanuts [11]. However, food allergens and the prevalence of FAs vary by geographical region. Family history, sex, ethnicity, genetic and molecular risk factors, environmental factors (including lifestyle disorders and consumption of more canned foods containing additives), and having other allergies such as asthma, rhinitis, and eczema are additional risk factors for the development of FAs [12]. Patients with FAs and asthma have a higher risk of developing fatal anaphylaxis than those without asthma [13-15].

Several studies have shown that the prevalence of FA in children and related emergency hospital visits is increasing in developed countries, especially in the last decade [16-19]. The prevalence of FA in all age groups is estimated to be approximately 8% [20]. The poor outcomes of FAs are primarily due to the limited management options and lack of curative therapy. Avoiding the offending food allergen and emergency treatment of any associated adverse reaction are currently the most common practices for the management of IgE- and non-IgE-mediated FAs [21]. The specific use of oral immunotherapy, which aims to induce tolerance to the relevant allergen in a controlled setting, is being considered a promising alternative treatment strategy for FA [8,22].

Due to limited epidemiological data on childhood FA in Saudi Arabia, this cross-sectional study aimed to estimate the prevalence and manifestations of parent-reported FAs among school children in Taif, Saudi Arabia, and to assess the relationship between FA and identify factors associated with it.

## Materials and methods

Study design and subjects

This observational, cross-sectional, survey-based study was conducted from July 2019 to December 2020. A total of 508 parents with children aged five to eight years studying in 17 selected kindergartens (n = 9) and primary schools (n = 8) from all regions in Taif city, Saudi Arabia were enrolled. The population size was calculated based on the extrapolated data from the 2019 General Authority for Statistics of the Kingdom of Saudi Arabia. The calculated population of children aged five to eight years in Taif city was approximately 106,468.

Data collection

Data were collected using a study-specific questionnaire that was reviewed by two pediatric allergists for content validity and modified accordingly. It was further reviewed by noncontent experts to assess the clarity of the questions, and a final version was adopted thereafter. The questionnaire was divided into the following three sections: the first part was focused on demographic data, for instance, the child’s age, sex, nationality, parents’ age, and parent's level of education. The second part contained questions related to the parents’ atopic history, and the third section had four questions related to the child’s health and FAs (Appendix). Written informed consent was obtained from all participants (parents or legal guardians of the children) before starting the study. The study was approved by the Research Ethics Committee of Taif University (approval number: 40-36-0175). For distributing the questionnaire to the selected schools in different regions of the city, approval was obtained from the General Directorate of Education in Taif city.

Statistical analyses

The data obtained were analyzed using the Statistical Package for the Social Sciences (SPSS) version 26 (IBM Corp., Armonk, NY). Categorical variables are presented as frequencies and percentages and the chi-square test (χ2) was used to compare them. All p-values < 0.05 were considered statistically significant. We modeled the data using generalized linear multivariate logistic regression analysis. We have used a full model with a term representing every single demographic variable as a predictor and "reported food allergy" as the only predicted variable. Validation was performed using residual analysis in terms of assessment of randomness, plotting residuals against fitted values and leverage, standardized residuals against fitted values, and calculation of deviance value.

## Results

Demographic characteristics of the study participants and children

Altogether, 508 parents participated in the study and completed the questionnaire with valid responses on behalf of their children. The demographic characteristics of the participants and their children are summarized in Table 1. Most children (34.3%) were aged eight years. The proportion of girls (60.6%) was higher than that of boys (39.4%), and 96.1% of these children were of Saudi nationality. Regarding parents, 48.6% of the fathers were aged 37-46 years and 45.3% of the mothers were aged 27-36 years. Approximately 48.2% of the fathers and 49.8% of the mothers had a bachelor’s degree.

**Table 1:** Demographic characteristics of the participants and their children The total number of participants (508) is used to determine the percentage.

Variable	No. (%)
Child’s age (years)	
5	109 (21.5)
6	110 (21.7)
7	115 (22.6)
8	174 (34.3)
Child’s sex	
Female	308 (60.6)
Male	200 (39.4)
Child’s nationality	
Non-Saudi	20 (3.9)
Saudi	488 (96.1)
Father’s age (years)	
26–35	107 (21.1)
36-45	247 (48.6)
46–55	126 (24)
>55	28 (5.5)
Mother’s age (years)	
<26	15 (3)
27–35	230 (45.3)
36–45	218 (42.9)
46–55	43 (8.4)
>55	2 (0.4)
Father’s education	
Primary school	28 (5.5)
Middle school	44 (8.7)
High school	164 (32.3)
Bachelor’s degree	246 (48.4)
Postgraduate degree	26 (5.1)
Mother’s education	
Primary school	45 (8.9)
Middle school	54 (10.6)
High school	133 (26.2)
Bachelor's degree	254 (50)
Postgraduate degree	22 (4.3)

Prevalence of parental-reported FAs and symptoms

Altogether, 30.5% (n = 155) of the surveyed parents reported that their children had an allergy. FA was the most common allergic disease among the children, with a prevalence of 16.1%, while 30.5% of the children had other atopic diseases, including eczema, asthma, allergic rhinitis, and drug allergy. Approximately 54.3% of the fathers and 26.4% of the mothers reported having an allergic disease, with allergic rhinitis being the most common type among the parents (11.6% and 14.6%, respectively). Meanwhile, FA was reported in only 1.8% of the fathers and 3.7% of the mothers (Table 2).

**Table 2:** Prevalence of reported allergies among the children and their parents The total number of participants (508) is used to determine the percentage.

Variable	No. (%)
Father’s allergy (n = 276)	
Present	276 (54.3)
Father's allergy type	
Allergic rhinitis	59 (11.6)
Asthma	23 (4.5)
Eczema	26 (5.1)
Food allergy	9 (1.8)
Drug allergy	1 (0.2)
Mother’s allergy	
Present	134 (26.4)
Mother's allergy type	
Allergic rhinitis	74 (14.6)
Asthma	15 (3)
Eczema	39 (7.7)
Food allergy	19 (3.7)
Drug allergy	9 (1.8)
Child’s allergy	
Absent	353 (69.5)
Present	155 (30.5)
Child's allergy type	
Food allergy	82 (16.1)
Eczema	71 (14)
Asthma	50 (9.8)
Allergic rhinitis	41 (8.1)
Drug allergy	14 (2.8)

The most common allergy-causing foods among the children with reported FAs were eggs (30.5%), peanuts (17.1%), and sesame (15.8%). Similarly, among all the studied children, the most common food allergen was found to be eggs (4.9%), followed by peanuts (2.7%) and sesame (2.5%) (Table 3). Altogether, 17.1% of the children with FAs reported allergies to more than one food item.

**Table 3:** Distribution of the studied children according to the type of food allergen as a percentage of children having food allergy and as a percentage of all studied children

Variable	No. (%)
Food allergens (in children with food allergy; n = 82)	
Eggs	25 (30.5)
Peanut	14 (17.1)
Sesame	13 (15.8)
Shrimp	9 (11.0)
Milk	9 (11.0)
Fish	9 (11.0)
Soybean	8 (9.8)
Wheat	4 (4.9)
Strawberry	4 (4.9)
Banana	4 (4.9)
Kiwi	3 (3.7)
Chocolate	3 (3.7)
Tomatoes	2 (2.4)
Cashew	2 (2.4)
Cherries	2 (2.4)
Fig	1 (1.2)
Beans	1 (1,2)
Blueberry	1 (1.2)
Date	1 (1.2)
Mango	1 (1.2)
Pistachio	1 (1.2)
Chicken	1 (1.2)
Eggplant	1 (1.2)
Food allergens (all children)	
Eggs	25 (4.9)
Peanut	14 (2.8)
Sesame	13 (2.6)
Fish	9 (1.8)
Shrimp	9 (1.8)
Milk	9 (1.8)
Soybean	8 (1.6)
Wheat	4 (0.8)
Strawberry	4 (0.8)
Tomatoes	4 (0.8)
Kiwi	3 (0.6)
Banana	3 (0.6)
Chocolate	2 (0.4)
Cashew	2 (0.4)
Cherries	2 (0.4)
Pistachio	1 (0.2)
Mango	1 (0.2)
Chicken	1 (0.2)
Eggplant	1 (0.2)
Date	1 (0.2)
Fig	1 (0.2)
Beans	1 (0.2)
Blueberry	1 (0.2)

Table 4 shows the most frequently reported symptoms of FA among the children, which included rash (41.5%), itching without rash (31.7%), and vomiting (22%).

**Table 4:** Prevalence of symptoms in children with a reported food allergy

Variable	No. (%)
Reported food allergy symptoms (n = 82)	
Urticarial rash	34 (41.5)
Itching without trash	26 (31.7)
Vomiting	18 (22)
Shortness of breath	16 (19.5)
Abdominal pain	13 (15.9)
Lips and tongue swelling	11 (13.4)
Diarrhea	7 (8.5)
Cough	1 (1.2)

Association between FAs in children and different factors

**Figure 1:**
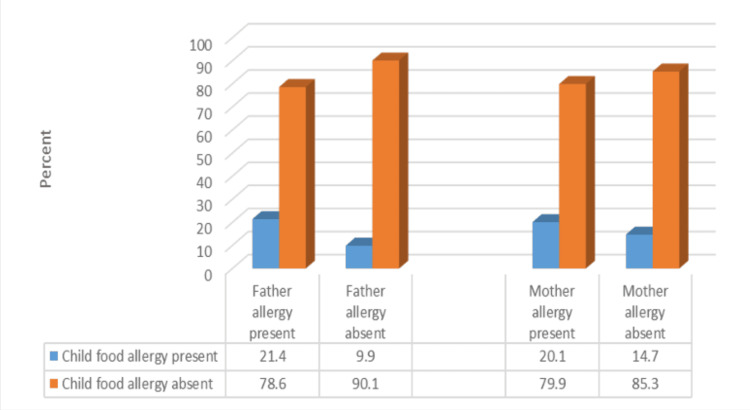
Association between the child’s food allergy and the mother’s or father’s allergy

The presence of parent-reported FA in a child was significantly associated with a positive allergy history of the father to asthma, allergic rhinitis, eczema, food allergy, or drug allergy (χ2 = 12.23; p < 0.001). However, we could not identify a significant association between reported FA in children and a history of allergy in their mothers (χ2 = 2.15; p = 0.14) (Figure 1).

Children aged five years (χ2 = 10.48; p = 0.015) or those who had any type of allergy (χ2 = 36.22; p < 0.001), allergic rhinitis (χ2 = 7.98; p = 0.005), or eczema (χ2 = 41.5; p < 0.001) had a significantly higher risk of having FA than the other children (Table 5). Conversely, the presence of FA in a child was not found to be significantly associated with the child’s sex, nationality, asthma or drug allergy, parents’ age, or parents’ allergies (p > 0.05; Table 5).

**Table 5:** Association of the presence of food allergy in a child with different variables

	Child’s food allergy			
Variable	Absent	Present	χ^2^	P-value
	No. (%)	No. (%)		
Child's age (years)				
5	81 (74.3)	28 (25.7)	10.48	0.015
6	94 (85.5)	16 (14.5)		
7	103 (89.6)	12 (10.4)		
8	148 (85.1)	26 (14.9)		
Child’s sex				
Female	265 (86)	43 (14)	2.74	0.097
Male	161 (80.5)	39 (19.5)		
Child’s nationality				
Non-Saudi	16 (80)	4 (20)	0.22	0.632
Saudi	410 (84)	78 (16)		-0.21
Father’s age (years)				
26–35	92 (86)	15 (14)	1.48	0.689
36–45	203 (82.2)	44 (17.8)		
34–55	106 (84.1)	20 (15.9)		
>55	25 (89.3)	3 (10.7)		
Mother’s age (years)				
<26	13 (86.7)	2 (13.3)	4.19	0.381
27–35	188 (81.7)	42 (18.3)		
36–45	185 (84.9)	33 (15.1)		
46–55	39 (90.7)	4 (9.3)		
>55	1 (50)	1 (50)		
Child’s allergy				
No	107 (69)	48 (31)	36.22	<0.001
Yes	319 (90.4)	34 (9.6)		
Child’s allergic rhinitis				
No	398 (85.2)	69 (14.8)	7.98	0.005
Yes	28 (68.3)	13 (31.7)		
Child’s asthma				
No	387 (84.5)	71 (15.5)	0.14	0.236
Yes	39 (78)	11 (22)		
Child’s drug allergy				
No	415 (84)	79 (16)	0.29	0.586
Yes	11 (78.6)	3 (21.4)		
Father’s food allergy				
Present	417 (83.6)	82 (16.4)	1.76	0.184
Absent	9 (100)	0 (0.0)		
Father’s other allergies				
Allergic rhinitis	53 (89.8)	6 (10.2)	1.75	1.85
Asthma	20 (87)	3 (13)	0.17	0.679
Eczema	24 (92.3)	2 (7.7)	1.44	0.229
Drug allergy	1 (100)	0 (0.0)	0.19	0.661
Mother’s food allergy				
Present	412 (84.3)	77 (15.4)	0.15	0.219
Absent	14 (73.7)	5 (26.3)		
Mother’s other allergies				
Allergic rhinitis	58 (78.4)	16 (21.6)	1.92	0.166
Asthma	10 (66.7)	5 933.3)	3.37	0.066
Eczema	32 (82.1)	7 (17.9)	0.1	0.75
Drug allergy	6 (66.7)	3 (33.3)	2	0.157

Table 6 presents the results of a multivariate logistic regression analysis of the risk factors for FA in children. It was found that having eczema and allergic rhinitis in childhood and the father’s history of allergy were significant and independent predictors/risk factors for a child’s FA (p < 0.05).

**Table 6:** Multivariate logistic regression analysis of the risk factors for food allergy in children Statistically significant values are in bold. BSc: Bachelor of Science.

	Estimate	OR	95% CI	P-value
Child's age	−0.159	0.853	0.685–1.062	0.154
Child's sex (male)	0.323	1.382	0.845–2.260	0.1975
Child's nationality (Saudi)	−0.301	0.74	0.228–2.405	0.6167
Father's age (years)	0.003	1.003	0.959–1.050	0.8916
Mother's age (years)	−0.017	0.983	0.937–1.032	0.4925
Mother's education (BSc)	0.121	1.129	0.670–1.902	0.6495
Father's education (BSc)	−0.132	0.877	0.522–1.471	0.6177
Father's allergy	0.772	2.164	1.261–3.712	0.0051
Mother's allergy	0.21	1.233	0.725–2.099	0.4394
Child's eczema	1.686	5.399	3.105–9.388	<0.0001
Child's allergic rhinitis	1.128	3.0903	1.406–6.7905	0.005
Child's drug allergy	0.22	1.2459	0.314–4.937	0.7543

## Discussion

FA has been investigated in children aged < five years and represents approximately 4.2% of FA incidence in all age groups of childhood [23]. However, there is no clear data on the exact prevalence of FA in Saudi Arabia. The population-weighted data obtained from the present cross-sectional, parent-reported survey estimated the prevalence of reported FA to be 16.1% among children aged five to eight years in Taif city in Saudi Arabia. A similar and recent study conducted in the eastern province of Saudi Arabia showed that approximately 34.4% of caregivers reported that their children had FAs and only 29.6% of them underwent confirmatory medical investigation [24]. Another study conducted in different areas of Saudi Arabia reported the prevalence of parent-reported FA in eastern, western, central, northern, and southern provinces to be 11%, 37%, 28%, 25%, and 25%, respectively [25]. The prevalence of FA worldwide is estimated at 6-13% [26]. A recent systematic review reported that 8.1% of children in the United Arab Emirates had FAs, with fish, eggs, and fruits being the most common allergens [27].

However, the actual prevalence of FA is difficult to establish as the data from previous studies are inconsistent due to several reasons, such as small sample size, focus on specific populations (not targeting the children) or only selected food allergens, or use of different protocols and definitions of FA. Moreover, self-reported FA prevalence is usually overestimated or higher than the actual physician-diagnosed prevalence, and the estimates confirmed by the gold standard of oral food challenges (OFCs), because of adverse food reactions, such as food poisoning, food intolerances, and enzyme deficiencies, being misinterpreted as FA [28,29]. In the United States, the prevalence of true FAs among children was reported to be 7.6% after excluding the data of those children whose parent-reported FA estimates were inconsistent with IgE-mediated FA data [30]. In a previous study, the prevalence of FA in Europe was found to be 17.3% and 5.9%, respectively, while the same study reported an overall pooled point prevalence of FA to be as low as 2.6% when FA was diagnosed based on clinical history or OFCs [31]. Using the same approach for defining FA, a Kuwait study estimated the FA prevalence in school children (aged 11-14 years) to be quite low at 4.1% [32], consistent with estimates from other reports using OFCs for the diagnosis of FA among children of the same age group [33,34].

Several latest research studies have delineated that in comparison to adults, children up to the age of 15 years are more likely to experience any type of allergies, including FAs [35,36]. According to our study findings, other than FA (16%), 30.5% of the children had atopic diseases, with eczema being the most common type (14%), followed by asthma (9.8%) and allergic rhinitis (8.1%). Previous epidemiological research has revealed that asthma and other allergy disorders may significantly overlap [37].

Regarding the foods that induced allergy among the children enrolled in the present study, eggs were the most frequently reported allergen, followed by peanuts and sesame, consistent with the findings of previous studies [25,38,39]. This predominance of eggs as a food allergen in childhood allergies has been observed in previous studies as well [25,40,41]. Peanut and egg allergies were present in up to 3% of children in the United States and 9.5% of Australian infants aged 11-15 months, respectively [42]. Other studies have further confirmed the food allergens reported in our study [24,25,43].

In this study, the most common FA signs were rash, itching without rash, diarrhea, and swelling of the lips and tongue. In a previous Saudi study, the most frequent symptoms were itching, swelling, and breathing alterations [28]. The commonly observed FA symptoms in the present study are comparable to those reported in previous studies on children [43,44].

Several risk factors leading to FA have been studied earlier and have been reviewed elsewhere [45,46]. The present study demonstrated a significant prevalence of FA in children (a) with eczema or allergic rhinitis, (b) children aged five years, and (c) children whose fathers had a history of allergy. FA is associated with coexisting asthma, rhinitis, and eczema due to IgE-mediated inflammatory mechanisms [47,48]. Compared with the prevalence of FA estimated in our study, prior studies have reported a higher prevalence of FA among children suffering from other atopic diseases, such as atopic dermatitis and allergic rhinitis [49-52]. Moreover, FA has been linked to asthma, and patients with FA and asthma have been found to have a higher risk of developing severe allergic reactions than those without asthma [53-55]. Additionally, a study conducted across allergy clinics in Saudi Arabia on 103 patients found that the prevalence of FAs was 6% among pediatric patients but only 1.5% among patients aged ≥14 years [56]. This explains why most allergies improve as the child grows.

Prior studies have established a link between FA and a family history of allergies, with participants with a positive family history of allergies having a higher risk of food allergies than those with no family history of allergies [57]. The influence of family history was also documented in an international study [58]. Additionally, food sensitization and allergy are more common in those with a first-degree relative who has a FA [59]. Moreover, monozygotic twins have a higher concordance of peanut allergy than dizygotic twins [60].

Limitations and strengths

The use of a self-reporting questionnaire could have a recall bias. The questions in this survey had a high degree of complexity and were required to effectively identify food allergies. The intricacy of the questions was necessary to gain a better understanding of the respondents' allergies. Moreover, the use of a cross-sectional study design may have revealed the association between a few variables without investigation of the causal relationships. However, survey-based approaches can help capture even those patients with FAs who may not have received formal physician-led diagnoses or evaluations.

## Conclusions

This study in Taif, Saudi Arabia investigated sources of food allergens and the prevalence of self-reported FAs, as well as identified significant risk factors among children. Having eczema or allergic rhinitis in childhood and the father’s history of allergy were found to be significant and independent risk factors for a child’s reported FA; however, this association may require further investigation. It highlighted the importance of health education programs to increase awareness, knowledge, and perception of FAs and to support those with the burden of FA management. The data will help clinicians and policymakers to establish efficient treatment and primary interventions for FA prevention.

## Appendices

**Table 7:** Study questionnaire

Questions	Options
First section: Epidemiological questions	
Child’s age (years)	
Child’s sex	A. Male
	B. Female
Child’s nationality	A. Saudi
	B. Non-Saudi
Father’s age (years)	
Mother’s age (years)	
Father’s education	A. Primary school
	B. Middle school
	C. High school
	D. Bachelor’s degree
	E. Postgraduate degree
Mother’s education	A. Primary school
	B. Middle school
	C. High school
	D. Bachelor’s degree
	E. Postgraduate degree
Second section: Parents’ health	
Does the father have any of the following diseases (either now or previously)?	A. Asthma
	B. Hay fever
	C. Eczema
	D. Food allergy
	E. Drug allergy
	F. None of the above
Does the mother have any of the following diseases (either now or previously)?	A. Asthma
	B. Hay fever
	C. Eczema
	D. Food allergy
	E. Drug allergy
	F. None of the above
Third section: Child’s health	
Does your child have any of the following diseases (either now or previously)?	A. Asthma
	B. Hay fever
	C. Eczema
	D. Drug allergy
	E. None of the above
Does your child have any food allergies? Immunoglobulin E (IgE)-mediated food allergy: An immune-mediated reaction that appears within minutes to a maximum of two hours after food ingestion	A. Yes
	B. No
Please report the possible food allergen associated with your child’s food allergy, if any.	A. Cow’s milk
	B. Egg
	C. Peanut
	D. Tree nuts
	E. Fish
	F. Shrimp
	G. Wheat (nonceliac wheat allergy)
	H. Soy
	I. Please specify if any other
Please specify any symptoms associated with the suspected food allergy.	A. Localized symptoms: itching, sting/burning of the lips/mouth or throat, urticarial rash, lip, and tongue swelling
	B. Abdominal symptoms: nausea, vomiting, crampy/colicky abdominal pain, diarrhea
	C. Respiratory symptoms: wheeze, stridor, watery rhinitis, redness of eyes/nose, cough, shortness of breath
	D. Skin symptoms: urticaria, itching, flushed skin
	E. Systemic reactions: anaphylaxis, syncope
	Urticarial rash may be differentiated from eczematous rash
